# Cyberbiosecurity: A Call for Cooperation in a New Threat Landscape

**DOI:** 10.3389/fbioe.2019.00099

**Published:** 2019-06-06

**Authors:** Lauren C. Richardson, Nancy D. Connell, Stephen M. Lewis, Eleonore Pauwels, Randy S. Murch

**Affiliations:** ^1^Merrick & Co., Arlington, VA, United States; ^2^Johns Hopkins Center for Health Security, Bloomberg School of Public Health, Baltimore, MD, United States; ^3^Wilson Center Science and Technology Innovation Program, The Wilson Center, Washington, DC, United States; ^4^Virginia Tech Research Center, School of Public and International Affairs, Virginia Polytechnic Institute and State University, Arlington, VA, United States

**Keywords:** biosecurity, cybersecurity, cyberbiosecurity, life sciences, bioeconomy, bioinformatics, synthetic biology, biomanufacturing

## Abstract

The life sciences now interface broadly with information technology (IT) and cybersecurity. This convergence is a key driver in the explosion of biotechnology research and its industrial applications in health care, agriculture, manufacturing, automation, artificial intelligence, and synthetic biology. As the information and handling mechanisms for biological materials have become increasingly digitized, many market sectors are now vulnerable to threats at the digital interface. This growing landscape will be addressed by cyberbiosecurity, the emerging field at the convergence of both the life sciences and IT disciplines. This manuscript summarizes the current cyberbiosecurity landscape, identifies existing vulnerabilities, and calls for formalized collaboration across a swath of disciplines to develop frameworks for early response systems to anticipate, identify, and mitigate threats in this emerging domain.

## Introduction

**Figure 1:**
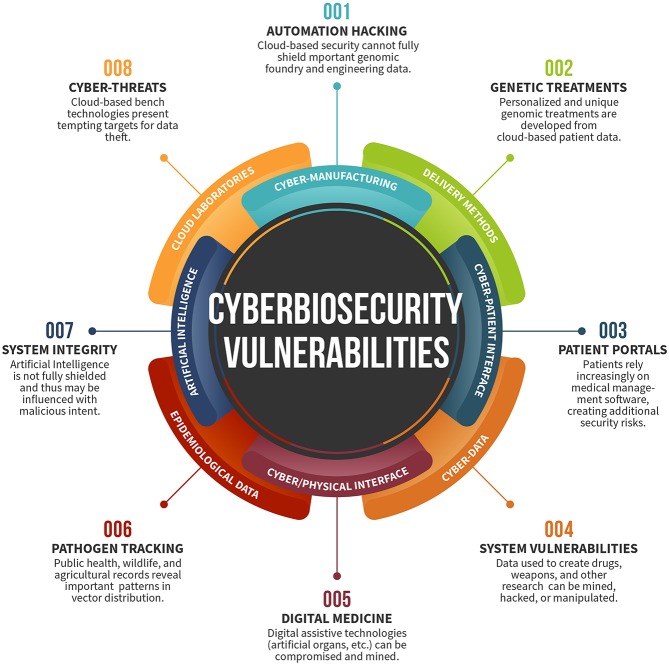
Summary of cyberbiosecurity vulnerabilities across the spectrum of the bioeconomy. Each sector of the wheel describes specific vulnerabilities that span the medicine, infectious disease, systems management, and biotechnology.

The greatest vulnerabilities in any field can be found at its margins—at its junctions with adjacent fields. The new discipline of cyberbiosecurity has been created to bring together disparate communities to identify and address a complex ecosystem of security vulnerabilities at the interface of the life sciences, information systems, biosecurity, and cybersecurity (Murch et al., 2018; Peccoud et al., 2018); it serves as a lens for observation that relies on disciplinary integration. Cyberbiosecurity describes an intersection of disciplines that falls outside any single sector; because these convergences are not clearly analyzed, actors within a single sector do not have agency to address potential issues and are less likely to cooperate. Such vulnerabilities exist within biomanufacturing, cyber-enabled laboratory instrumentation and patient-focused systems, “Big Data” generated from “omics” studies, and throughout the farm-to-table enterprise (Figure 1). In addition to fundamental and applied research and development opportunities, off-the-shelf solutions not yet applied in this domain likely exist. While the term is new, the concept of cyberbiosecurity has been acknowledged as a serious concern (Wintle et al., 2017). The issues raised in the area of cyberbiosecurity will have substantial impact on the growing bioeconomy^1^.

The solution set is not simply technical: creating cross-sector convergence opportunities for effective communication and collaboration as well as governance, policy, and regulatory structures is also necessary. Derived value from cyberbiosecurity endeavors potentially embraces economic impact, national security, societal resilience, and environmental sustainment. In this paper, we establish a landscape for cyberbiosecurity and issue a call for cooperation across sectors to recognize and mitigate potential threats.

## Background

As a part of the discussion, we refine the definition of cyberbiosecurity. **Cybersecurity** encompasses the protection of computer systems from theft and damage to their hardware, software, or information, as well as from disruption or misdirection of the services they provide. **Biosecurity** involves securing valuable biological material from misuse or harm. Initially, Murch et al. defined cyberbiosecurity as the “developing understanding of the vulnerabilities to unwanted surveillance, intrusions, and malicious and harmful activities which can occur within or at the interfaces of comingled life science, cyber, cyber-physical, supply chain and infrastructure systems, and developing and instituting measures to prevent, protect against, mitigate, investigate, and attribute such threats as it pertains to security, competitiveness, and resilience” (Murch et al., 2018). The definitions of cybersecurity and biosecurity both include an underlying assumption of value on the part of the material in question. We further suggest expansion of this definition of cyberbiosecurity to differentiate it from the individual scopes of cybersecurity and biosecurity. Cyberbiosecurity addresses the potential for or actual malicious destruction, misuse, or exploitation of valuable information, processes, and material at the interface of the life sciences and digital worlds; concept mastery requires an understanding of this interface in the context of the threat of malignant use of technology in general. This paper is a call to action before such a succession of events takes place.

## Landscape

Cyberbiosecurity cuts across disciplines; impacting fields from laboratory science, to human and animal health, agriculture, and environmental health and ranging from protection to management and remediation. Technology integration is the new norm, with novel technology improvements and simple digitization bringing easy access to old systems, such as medical records. As technical disciplines develop at an exponential pace and their convergence accelerates, it is becoming increasingly clear that the fields of cybersecurity and biosecurity must also converge in order to address inherent digital and biological concerns. Further, technological convergence meets the decreasing cost for access at the Do It Yourself (DIY)/community biology space.

## Cyberbiosecurity in Biotechnology

### Artificial Intelligence

Industry interest in artificial intelligence (AI) has experienced a resurgence in recent years due to increased computing power, advancing applications of neural networks, and an emergence of new machine and deep learning techniques across the biology sector. Biotechnology companies are successfully utilizing these developments for drug design and development (Zilinskas, 2017), genomics (Pauwels and Vidyarthi, 2017), evolutionary biology (Feltes et al., 2018), protein folding (Paladino et al., 2017), and more. This rapid and evolving interest in the landscape of new AI technologies has led to emerging threat domains related to information privacy and storage, ownership over biological and genetic data, and applications of powerful technologies (Pauwels, 2018). These issues are not new, as bioinformatics and digitization have created a potential target; however, the popularization of AI has refreshed these concerns in the modern zeitgeist. There is a renewed opportunity for life science and cybersecurity professionals to design and implement frameworks to facilitate responsible application of AI techniques to biology.

### Automation

The convergence of robotics, machine learning, and artificial intelligence has paved the way for automated approaches to biology, manufacturing, software development, accounting, and more. Improved biological engineering techniques and robotics have converged to result in rapid prototyping and higher yields. Laboratories are increasingly using robots to improve throughput and free up the hands of laboratorians around the world (McGee, 2014; Szesterniak, 2014). As robots are increasingly connected to networks and other electronic systems, new cyberbiosecurity concerns unique to automated laboratory environments are beginning to emerge. Virtual environments allow access to infrastructure within the physical world; this creates a vulnerability that would permit unauthorized remote access to an automated biological manufacturing system. As automation increases within the life sciences, so too will potential vulnerabilities to threat.

### Synthetic Biology

The term “synthetic biology” is widely used to describe activities carried out by scientists in a variety of disciplines, from bioengineering, chemistry, biochemistry, and materials science to cellular and molecular biology (Hobom, 1980; Purnick and Weiss, 2009). Today, engineers, biologists, technologists, and citizen scientists have turned this field into a true discipline. Systems engineering techniques are being applied to organisms to design genetic circuits, novel molecules, and commodities such as fuels, electricity, feed, and renewable materials (Rollin et al., 2013; Kiss et al., 2014). Simultaneously, the design-build-test approach traditionally used in product development is rapidly emerging in organism engineering (Dudley et al., 2015; Gill et al., 2016). Advancements in synthetic biology will have a significant impact on cyberbiosecurity as laboratory automation techniques become more widespread and the traditional cost barrier for scale-up of production is lowered. Similarly, the convergence of robotics, microfluidics, cell-free systems design and synthetic metabolic engineering stands to create new cyberbiosecurity risks and unique threat domains (Nielsen and Keasling, 2011; Murch et al., 2018; Peccoud et al., 2018). As these fields further develop and converge, revealed vulnerabilities will offer new opportunity for exploitation.

## Cyberbiosecurity in Digitization of Traditional Technology

### Manufacturing

Science and technology-reliant organizations are becoming more complex and networked throughout facilities, supply chains, logistics, and transport mechanisms. Distributed manufacturing employs decentralized production networks linked by information technology; as more connections between traditionally isolated systems are developed, more security controls must be considered in order to mitigate risks and reduce vulnerabilities. The production processes and assemblies of biologics and other materials can also be distributed and carried out asynchronously at geographically different locations, allowing response to potential threats to be developed *in situ*.

In addition to facilitation of distributed manufacturing techniques for traditional life sciences operations, recent advances in cell-free metabolic engineering technologies allow for higher throughput in production environments. This has resulted in improved biological techniques for rapid prototyping and higher yields. Cell-free biological systems are being used to develop commodities such as fuels, electricity, feed, and renewable materials (Rollin et al., 2013). As the convergence of dichotomous technical disciplines (e.g., automation and cellular biology) continues to expand rapidly, it is increasingly important that the fields of cybersecurity and biosecurity converge to address inherent digital and biological concerns.

### Biomedical Sciences

Cybersecurity and health security converge with increasing digitization of health data. Regulatory mechanisms are in place to address concerns regarding privacy and confidentiality of medical and billing information; however, this extends beyond the cyber-patient interface in the context of electronic medical records. Patient treatment management—including potential drug interactions, protocols, and sensitivities specific to the patient—is increasingly digitized. Personalized medicine diagnostics and therapeutics are rapidly expanding, and much of the information associated with these interventions is maintained digitally. Biomedical data breaches are not without historic precedent: in 2014, data breaches of three major health systems resulted in unauthorized access to millions of patient records, including clinical data (Kozminski, 2015). These breaches provided the perpetrators valuable clinical data, which could be used internally or sold for monetary gain. In addition to facilitating illicit data collection, disruption of digitally-programmed diagnostic testing systems or therapeutic targeting fields could result in ineffective treatment. Medical devices are also an area of interest in cyberbiosecurity, as many potential exploits could be leveraged through direct and indirect interfaces with the patient and manufacturer (Khera, 2017).

### Agriculture

Throughout much of the world, food and beverage safety and security is a high priority. Concomitantly, the economics, societal robustness, and security implications of agriculture, foodstuffs and beverages are massive. Extensive quality measures are in place to prevent and mitigate threats from manifesting; outbreak and contamination detection and response systems react when problems are noticed. Packaging and labeling methodology have also been improved. However, agriculture and consumables in many countries rely on cyber-enabled systems for many aspects of farm management, production-to-consumption, raw materials to finished product, and logistics (Security Security DoH., 2018). The health and security of this dimension of agriculture and food systems is unclear from a cyberbiosecurity perspective. We reason that vulnerable critical links and nodes exist throughout this highly complex global and national ecosystem; attention to cyberbiosecurity measures is warranted and would be considerably beneficial.

## Conclusion

The convergence of recent advances in the life sciences with regard to traditional cybersecurity threats has led to the recognition and identification of vulnerabilities, known as cyberbiosecurity threats (Murch et al., 2018; Peccoud et al., 2018). Here we present a preliminary review of the landscape of these threats and propose recommendations to activate a “call to action” to anticipate these threats and mitigate their effects. Several entities have approached related issues: for example, in October 2019, HHS announced the opening of the Health Sector Cybersecurity Coordination Center (HC3), intended to prevent threats to health data through strengthening cybersecurity (Office Office HP., 2018). Though concurrent efforts touch on the issues described, individual efforts alone are insufficient to cover the breadth of the landscape. We call for analyses and publications to fully scope cyberbiosecurity and identify a comprehensive strategy to establish the discipline's goals and objectives; we call for carefully-crafted national or international meetings of experts from appropriate science, technology, and social science domains to begin to bring communities together to define priorities for approaches to solutions by examining causes, effects and possible remedies; we call for initiation of campaigns of blended teams of experts engaging key government agencies to raise awareness and initiate creation of and/or changes to relevant policies and programs in order to incorporate relevant cyberbiosecurity perspectives.

## Author Contributions

LR, NC, SL, EP, and RM contributed conception and design of the manuscript. LR, NC, SL, and RM wrote sections of the manuscript. All authors contributed to manuscript revision, read, and approved the submitted version.

### Conflict of Interest Statement

LR and SL were employed by Merrick and Company. The remaining authors declare that the research was conducted in the absence of any commercial or financial relationships that could be construed as a potential conflict of interest.

## Footnotes

^1^Bioeconomy is defined as “economic activity that is fueled by research and innovation in the biological sciences (House, 2012).”
